# The Role of Population Origin and Microenvironment in Seedling Emergence and Early Survival in Mediterranean Maritime Pine (*Pinus pinaster* Aiton)

**DOI:** 10.1371/journal.pone.0109132

**Published:** 2014-10-06

**Authors:** Natalia Vizcaíno-Palomar, Bárbara Revuelta-Eugercios, Miguel A. Zavala, Ricardo Alía, Santiago C. González-Martínez

**Affiliations:** 1 Department of Forest Ecology and Genetics, Forest Research Centre (INIA), Madrid, Spain; 2 Department of Life Sciences, University of Alcalá, Alcalá de Henares, Madrid, Spain; 3 Centre for Economic Demography, Lund University, Lund, Sweden; 4 Sustainable Forest Management Research Institute, University of Valladolid-INIA, Palencia, Spain; 5 Department of Ecology and Evolution, University of Lausanne, Lausanne, Switzerland; INRA - University of Bordeaux, France

## Abstract

Understanding tree recruitment is needed to forecast future forest distribution. Many studies have reported the relevant ecological factors that affect recruitment success in trees, but the potential for genetic-based differences in recruitment has often been neglected. In this study, we established a semi-natural reciprocal sowing experiment to test for local adaptation and microenvironment effects (evaluated here by canopy cover) in the emergence and early survival of maritime pine (*Pinus pinaster* Aiton), an emblematic Mediterranean forest tree. A novel application of molecular markers was also developed to test for family selection and, thus, for potential genetic change over generations. Overall, we did not find evidence to support local adaptation at the recruitment stage in our semi-natural experiment. Moreover, only weak family selection (if any) was found, suggesting that in stressful environments with low survival, stochastic processes and among-year climate variability may drive recruitment. Nevertheless, our study revealed that, at early stages of recruitment, microenvironments may favor the population with the best adapted life strategy, irrespectively of its (local or non-local) origin. We also found that emergence time is a key factor for seedling survival in stressful Mediterranean environments. Our study highlights the complexity of the factors influencing the early stages of establishment of maritime pine and provides insights into possible management actions aimed at environmental change impact mitigation. In particular, we found that the high stochasticity of the recruitment process in stressful environments and the differences in population-specific adaptive strategies may difficult assisted migration schemes.

## Introduction

Sustained tree recruitment is fundamental to ensure forest persistence under global climate change [1], [2]. Understanding tree recruitment processes and potential biotic and abiotic interactions can help to realistically forecast the distribution of future forests. Tree recruitment involves multiple life-history stages –from seed to adult tree– that are connected by transitional processes: seed maturation, dispersal, germination, emergence and survival. Demographic collapse at any of these stages can doom recruitment [3], [4], jeopardizing a long-term preservation of the population. Many studies have reported relevant ecological factors that affect recruitment success in trees –light, microtopography, physical and chemical soil characteristics, herbivory, pathogens and competition with herbs [5]–[7]– but the potential for genetic-based differences in recruitment (among populations and families of the same species) has often been neglected in experimental or modeling studies. For example, in Mediterranean environments, summer drought and the shortness of favorable periods (i.e. when temperature and water availability are suitable for plant growth) are main abiotic factors constraining tree establishment [1], [8], [9]. It is also well-known that Mediterranean tree populations and families have large genetic differences in drought response and growth [10]–[13]. However, genetic factors are rarely considered in Mediterranean tree recruitment studies.

From a demographic standpoint, the seed and seedling stages are probably the most vulnerable [2], [14]. This is typically reflected in a low early survival, with one-season mortality rates up to 70–100% [1], [2], [14]–[16]. High mortality rates in seedlings are mainly related to their small size, which makes them susceptible to factors that generally affect larger plants only to a lesser extent, such as competition with neighboring vegetation (including intraspecific competition), browsing, extreme climatic events, and insect or disease infestation [14], [17]. These biotic and abiotic ecological factors, among others, bring about strong selective pressure that is expected to affect population genetic structure within and among populations, in turn influencing adult traits and fitness [18], [19].

Natural selection can cause evolutionary change on contemporary time scales (e.g. [20]) and, by filtering the better adapted genotypes, natural selection can result in local adaptation [21]. Many forest tree species are thought to be locally adapted (reviewed in [22]), in particular following environmental clines along large geographical scales [23]–[25]. The existence of past local adaptation in forest trees at the population level may indicate some capacity to face new pressures arising from climate change (e.g. bark beetle outbreaks caused by warming conditions [26]), although, at the same time, those locally-adapted genotypes may not be able to respond to unprecedented rapid warming due to genetic constrains [25], [27], [28].

Reciprocal transplant experiments are a powerful way of testing for local adaptation (sensu Kawecki and Ebert, i.e. the relative fitness of local and foreign populations between sites or habitats [29], [30]). There is an ample literature on local adaptation using reciprocal transplant experiments (see reviews in [31], [32]) that has shown that local populations often have a higher fitness than non-local ones, with more significant differences detected when population sizes are big [31]. Typically, local adaptation studies have focused on fitness-related traits such as reproductive success, plant size and survival rates, usually under controlled conditions across contrasted sites or along gradients. However, they have rarely considered long-lived plant species such as forest trees, or the early stages of establishment when natural selection is expected to be stronger.

The early stages of plant life are time- and microenvironment- dependent [33]–[35]. Temporal and environmental heterogeneity is large in Mediterranean woodlands, even within the same forest [2]. Plant establishment success largely depends on the timing of key regeneration processes, such as dispersal and germination. With respect to microenvironmental factors, the light regime, governed by tree canopy cover, is probably the most important in Mediterranean forests [36], [37]. A partial canopy cover may protect seedlings from high radiation and temperatures and from losses of soil moisture in stressful sites, thereby increasing early survival [9], [37]. This is particularly relevant in species that recruit in summer, such as Mediterranean pines. Additionally, as summer develops, heat cumulative degrees make open-canopy areas less favorable for emergence in comparison to sites with partial shade. Therefore, a shift in emergence from open-canopy to shaded areas is expected.

Maritime pine is a typical component of Mediterranean landscapes. This emblematic pine is characterized by a scattered distribution that may have both limited gene flow among different groups of populations, and high levels of genetic divergence promoted as a result of combining genetic drift and natural selection (*Q_ST_* = 0.29–0.46 for height growth and survival, Isabel Rodríguez-Quilón per. comm.; see also [38]). At the phenotypic level, water availability and forest fires are considered to be major drivers of local adaptation in maritime pine [10], [39]. However, among its literature (e.g. [36], [40]), there are no studies considering the species' genetic make-up at the early stages of establishment in natural populations. The aim of this work is to assess the factors controlling emergence and early seedling survival in natural maritime pine forests and in particular, the role of local adaptation and microenvironment. To accomplish this general goal, we established a semi-natural reciprocal sowing experiment in the field under distinct canopy-cover (i.e. light regime) conditions using two contrasted maritime pine origins that have evolved under different selective pressures associated with drought response and fire regime: Coca (central Spain), a non-fire adapted, continental-dry population living in sandy soils, and Calderona (eastern Spain), a population that is subjected to milder climate and adapted to fire [41].

Most reciprocal transplantation experiments are based on spaced plantings of seedlings previously produced in greenhouse conditions. This may limit some of the effects of natural selection at the germination and early establishment stages (e.g. [42], [43]). To circumvent this issue, we developed a novel approach that avoided greenhouse manipulation and used molecular markers to identify the genetic origin (i.e. the family) of the surviving seedlings. We tested for local adaptation (following [29], [31]), considering two distinct microenvironments relevant for tree recruitment and forest colonization: open-canopy (i.e. under light exposition) vs. closed-canopy (i.e. under partial shade by dominant trees). The interactions between local adaptation at different genetic levels (population and family) and microenvironment (i.e. environmental heterogeneity in light regime within populations) have never been studied before in semi-natural conditions and may provide new insights into how genetic adaptation takes place in natural plant populations.

Using our semi-natural reciprocal sowing experiment, we specifically addressed the following questions: (i) Is there local adaptation for emergence and early survival in maritime pine populations of contrasted origin?; (ii) Is early survival affected by time of emergence?; (iii) What is the role of canopy cover (i.e. light regime) in this species' emergence and early survival?; (iv) Can we observe a transition in emergence and survival from open- to closed-canopy environments along the summer season?; and (v) Is natural selection acting equally on all families within populations or, alternatively, is natural selection favoring some families, resulting in genetic change over generation for heritable traits? Our expectation is that, despite range fragmentation, maritime pine populations may have maintained enough standing genetic variation for local adaptation and family selection to have taken place, which would explain the large phenotypic variability observed across natural populations of this Mediterranean conifer.

## Materials and Methods

### Ethics Statement

Permits to conduct research (including plant material collection) in Coca (latitude: 41°15′17″N, longitude 4°29′52″W) and Calderona (latitude: 39°44′56″N, longitude 0°29′44″W) sites were obtained from the Forest Services of the Autonomous Communities of ‘Castilla y León’ (Contact Person: Ing. María Bragado) and ‘Valencia’ (Contact Person: Mr. Antoni Marzo), respectively. These field studies did not involve endangered or protected species or have any long-term consequences for the forests studied.

### Study sites and experimental design

**Figure 1:**
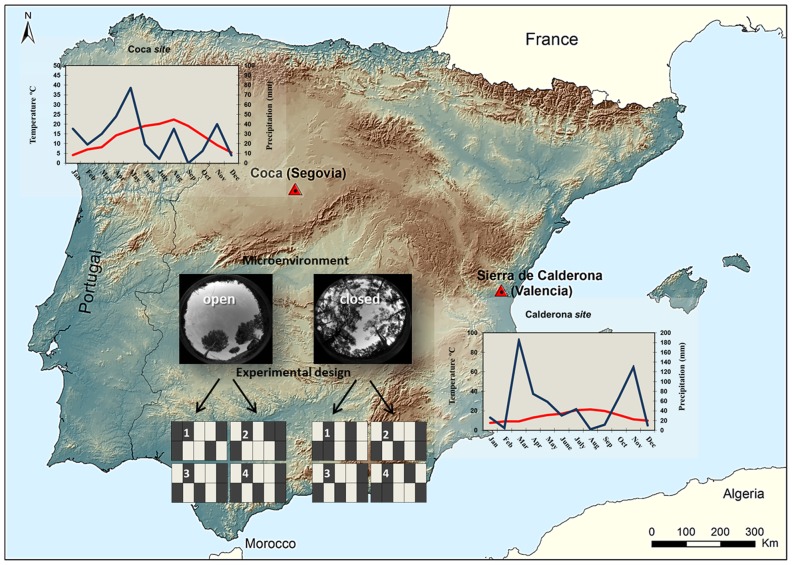
Semi-natural reciprocal sowing experiment established in maritime pine to study local adaptation at early stages of establishment. Experimental sites and population's origin are shown in red triangles. Climodiagrams (mean monthly temperature in red and monthly precipitation in blue) of both sites are also given (data from the Spanish National Meteorological Agency, after corrections using Gonzalo's phitoclimatic model for Spain [44]). The experimental design (in the center of the figure) consisted in a split-plot with four replicates in each microenvironment (open canopy, exemplified in the photo on the left side; and closed canopy, the photo on the right side); dark grey and light grey boxes represent the two origins tested (Coca and Calderona).

Our reciprocal sowing experiment was conducted in two Mediterranean maritime pine (*Pinus pinaster* Aiton) sites separated by a distance of 535 km (Figure 1). The first site was located in Coca, in the Spanish province of Segovia, situated in the Castilian central plateau (from now on “Coca *site*”). Its mean and maximum daily air temperatures over the course of the experiment, from March to September 2011, were 17.15 and 25.09 °C, with a range of 8.20–22.32 and 13.69–30.86 °C, and coefficient of variation, C.V., of 0.32 and 0.28, respectively (data from Migueláñez meteorological station, c. 18 km away from the study site, corrected using Gonzalo's phitoclimatic model for Spain [44]). Annual precipitation and precipitation during the study period (March to September) were 329.01 mm and 214.36 mm, respectively. Partial records in a climate logger from May to September (HOBO Weather Station Data Logger Massachusetts, USA) showed similar mean soil daily temperatures, but much lower C.V. (0.11) than air temperatures (Figure S1), and relative humidity of 49.44% (range of 17.25–86.53%, C.V. of 0.19). The second site was located in Sierra de Calderona in the Spanish province of Valencia (from now on “Calderona *site*”) in the eastern Iberian coastal mountains. Its mean and maximum daily air temperatures over the course of the experiment were 16.77 and 22.83 °C, with a range of 9.14–21.58 and 13.97–28.16 °C, and C.V. of 0.27 and 0.24, respectively (data from Segorbe meteorological station, c. 6 km away from the study site, corrected using Gonzalo's phitoclimatic model for Spain [44]). Annual precipitation and precipitation during the study period (March to September) were 643.80 mm and 405.70 mm, respectively. As in the case of Coca, an in situ climate logger (HOBO Weather Station Data Logger Massachusetts, USA) recorded similar but less variable (C.V. of 0.08) soil than air temperatures (Figure S1). Relative humidity was 63.87% (range of 37.92–87.18%, C.V. of 0.26). A comparison of climatic data from the study period with historical data recorded between 1983 and 2013 indicated that the study year was not far from historical records, except for a somewhat warmer April (see details in Figure S2). Overall, Calderona *site*, with slightly lower temperatures (in particular maximum temperatures) but much higher precipitation (both annual and during the duration of the experiment) and relative humidity, presented a less extreme climate when compared to Coca *site*. Temperature variability (evaluated by C.V.) was also higher in Coca *site*. Besides, the two study sites differed in soil substrate. Calderona *site* presented a developed substrate composed by litter and leaves from typical Mediterranean shrubs; whereas Coca *site* presented a sandy soil, with c. 90% of sand (see soil analysis in [36]), which limits its capacity to retain rain water.

The experimental design was a complete randomized and balanced split-plot, where each plot was composed by 10 subplots, five for each population origin (Figure 1). Dimensions of plots were 6 m×5 m, and dimensions of subplots 1 m×2.5 m. A narrow corridor of 20 cm was left between subplots to ease sowing during experiment establishment, and to allow both monitoring of seedling emergence and death events without disturbing natural recruitment during experiment duration. Plots were physically divided using a thin rope. To avoid predation by birds and small mammals, plots were protected and covered with 2.5×3 cm gardening mesh (PROJAR S.A., Spain). This large mesh size was chosen to avoid any significant modification of microenvironmental conditions out and under the mesh. To ensure similar initial conditions in both experimental sites and avoid facilitation/competition effects, shrubs (mainly *Quercus coccifera* and *Ulex* sp.) were removed using pruning scissors before sowing when needed (Figure S3). We tried not to manipulate the soil surface or to remove litter in any of the two field sites not to change soil conditions for emergence.

To test for differences in local adaptation related to environmental heterogeneity within each experimental site, plots were placed under either open or partial shade canopy (i.e. ‘closed canopy’), as light regime is a major factor determining soil microhabitats and has a relevant impact on pine survival and establishment [40]. Light availability was estimated by means of hemispherical canopy photography. Photographs were taken at the seedling level in the centre of each experimental unit, using a horizontally levelled digital camera (Coolpix 4500, Nikon, Tokyo, Japan) with a fish-eye lens of 180° view field (FCE8, Nikon). We used Hemiview Canopy Analysis v. 2.1 software (Delta-T Devices Ltd, Burwell, UK) to analyze the images and calculate Global Site Factor (GSF), which represents the fraction of total radiation above the canopy that penetrates below the canopy [45]. Open-canopy plots, GSF values of 0.72±0.05 (mean ± SE), represented areas where future seedlings would be more free from the influence of adult trees, in particular shadowing, on bare soil with areas of sparse herbaceous vegetation (mainly annuals and short-lived perennials), whereas closed-canopy plots, less accessible for light with GSF values of 0.51±0.04, were placed in areas under much more direct adult-tree influence. Four replicates were established on each of the two canopy cover classes (see Figure 1).

### Plant material

Unlike most reciprocal transplant experiments, ours was based on random sowing of seeds and not on planting greenhouse-raised seedlings or regularly-spaced seeds either on the soil or in pots. This strategy was chosen for two reasons: (i) to fully consider selection pressure taking place at germination, emergence and early establishment, including intra-specific competition, and (ii) to avoid seedling mortality due to transplant shock that may be confused with natural selection.

Needles and mature female cones from 25 individuals (i.e. maternal trees) were collected from each population (Coca *origin* and Calderona *origin*) at about the same date in February 2011. Needles were kept in individual paper bags inside a bigger plastic bag filled with silica gel. This material was used to obtain mother-tree genotypes using molecular markers (see below) and to be able to assign each offspring to its family at the end of the experiment. In this way, seeds from different families could be pooled without risk of losing their family identity or modifying their natural emergence and first establishment conditions. Mature female cones were collected to extract the 1,600 seeds per family (see below) needed for our experiments. To open the cones and extract the seeds, they were placed in a forced-air oven at 60 °C for approximately 48 h. Then, seed wings were removed from seeds manually with a winnow. We prepared 80 samples (5 subplots×8 replicates×2 experimental sites) of 20 seeds per family from each of the two sites (hereafter Coca *origin* and Calderona *origin*), i.e. a total of 160 seed lots. Average seed weight did not significantly differ between populations (Calderona *origin*: 0.054±0.002 g (mean ± SE) and Coca *origin*: 0.062±0.003 g). The whole experiment involved 80,000 seeds, 40,000 (1,600 seeds per family×25 families) from each origin. Finally, at the end of the experiment, surviving seedlings were collected and kept in collection microtubes with silica gel until DNA extraction.

### Data collection

In maritime pine, dispersal begins in the spring and peaks during the summer months (July and August), often associated to dry episodes or storms [46]. Sowing took place in 2011, on March 10^th^ in Coca and April 7^th^ in Calderona, therefore it approximately coincided with the beginning of the year's dispersal. Seeds were thrown randomly along the longest side of each subplot simulating natural dispersion as much as possible. The experiment was visited approximately every 20 days. On each visit, the date of every new emergence was individually tagged and recorded as well as any mortality events. We did not to register new emergences from July 1^st^ onwards to avoid the monitoring of naturally-dispersed seeds from local pines. Competition intensity was not strong, as judged by relatively sparse germination (see Figure S4), and most seedling mortality apparently took place due to seasonal drought. The end of the experiment took place on September 20^th^. Raw data is provided as Data S1.

### Molecular markers

Needles from the mother trees (*N* = 50) and all surviving offspring in the reciprocal sowing experiment (*N* = 56, see Results) were collected and dried in silica gel for subsequent DNA extraction using the Invisorb DNA Plant HTS 96 Kit/C kit (Invitek GmbH, Berlin, Germany). Four high-resolution (Polymorphic Information Content, PIC, of 0.488–0.822, and combined Exclusion Probability, EP, of 0.91, see [47] for a definition of EP) nuclear microsatellites (nuSSRs) were scored in all samples (total *N* of 106), except mother trees from Coca *origin* that were scored with only three (NZPR1078 did not amplify; combined EP of 0.87), following protocols in [47]: ITPH4516 and FRPP94, and Chagné et al. [48]: NZPR1078 and NZPR413. PCR fragments were resolved on a Li-Cor 4300 DNA analyser (Li-Cor Biosciences, Nebraska, USA). To reduce the probability of scoring errors, a selection of samples that covered the fragment size range was included as internal standard in each gel. SAGA^GT^ vs. 3.3. was used for gel calibration and scoring (Li-Cor Biosciences, Nebraska, USA).

### Temporal patterns of emergence and early survival

We first examined the evolution of seedling emergence and early survival with time for each of the maritime pine origins, considering the two experimental sites (Coca vs. Calderona) and microenvironments (open canopy vs. closed canopy), as well as the interactions between these factors. For this purpose, we conducted a non-parametric survival analysis appropriate for duration data (see review in [49]). Our survival analysis can handle right censored data properly (avoiding biased results) and does not require normally distributed data [50], [51]. Emergence and survival data are right censored because many individuals either did not emerge or were still alive at the end of the observation. We computed the non-emergence and survivor functions, NE(*t*) and S(*t*) to measure, respectively, the probability of a seed of staying in the ground or a seedling of surviving (among those that emerged) beyond time *t*: NE(*t*) or S(*t*) = P[*T*≥*t*]. We used the Kaplan-Meier (KM) estimator, which provides empirically constructed and discrete step functions over time in the different cases considered. The Kaplan–Meier estimator, 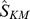, for non-emergence and survival is given by:
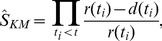
(Eq.1)where *d*(*t_i_*) is the number of emergences or deaths at time *t_i_* and *r*(*t_i_*) is the number of individuals at risk at time *t_i_* (i.e. those that have not emerged or died yet). We computed Kaplan-Meier estimators over time for each combination of sites and microenvironment (i.e. canopy-cover class). To assess statistical differences between functions we used log-rank tests that are suitable when censored data is present [52]. All analyses were performed with the *survival* package ver. 2.37–4 [53] in the R environment [54].

### Local adaptation and the role of the microenvironment

To provide insights into local adaptation patterns at the early stages of establishment in maritime pine, we used discrete-time logistic models within the survival analysis framework. This approximation has been seldom used to address ecological problems but it is widespread in biomedical and sociological sciences [55], [56]. Alternative approaches, such as accelerate failure models (AFT) or proportional hazard models (Cox), treat time as continuous, which in our case would have been incorrect since we observed recruitment processes in discrete units of time. Moreover, taking time as continuous would have forced us to assume a determinate shape for the emergence and survival time distributions (i.e. exponential, gamma, lognormal, etc.), which, given few observation points along time, could have led to variable results depending on function specification [55]. Finally, continuous-time approaches are not convenient when there is a large amount of ties (i.e. simultaneous occurrence of an event for many individuals), as in our case.

Therefore, instead of measuring the time to failure, we modeled each interval between censuses individually, by creating a binary variable, 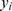 (0, 1), which indicates whether the event (emergence or survival) occurred in the interval [t, t+1]. With this method, one record for each interval time for each individual is obtained. In practical terms, this increases the number of observations but it does not produce any bias [55]. The probability that individual *i* emerges or survives during interval *t*, 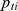, given that no event has occurred before the start of *t*, can then be computed as:



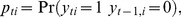
(Eq.2)

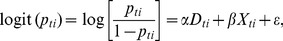
(Eq.3)where 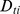 is the vector of coefficients α of the step function that captures the baseline hazard function, i.e. the risk per interval time that an event happens (as it is called in continuous time analysis). Four and five intervals were considered in the emergence and survival models, respectively, according to visit number to the field sites. The 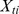 is a vector of explanatory covariates and *β* the corresponding estimated coefficients.

In our analysis of emergence and early survival, we assessed the following variables: (i) experimental site (Calderona and Coca), (ii) population's origin (Calderona and Coca), (iii) microenvironment (open and closed canopy), (iv) all two-way possible interactions of the latter (in particular interaction between site and population origin, which can be considered a formal test of local adaptation), and (v) census (time) interval. In addition, in the early survival analysis, time to emergence was included as another independent covariate. In both models, Calderona *origin*, Calderona *site* and closed-canopy microenvironment were chosen as the reference level for the odds ratios. For census intervals, odds ratios offer a picture of the probability of emerging or surviving for each of the intervals between censuses, controlling for the rest of the effects. To obtain robust standard errors despite repetitive measures of the same individuals (mainly associated with the 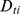 vector), the discrete-time logistic analyses were clustered around individuals [57]. Coefficients in emergence and survival models were estimated using maximum likelihood. Statistical significance was assessed by Wald χ^2^ test [58]. Analyses were performed using Stata vs. 11 [59].

Finally, as a complementary method to discrete-time logistic models, we also computed effect sizes (Hedges' g*, i.e. a standardized and unbiased estimate of mean differences [60], [61]) for local and microenvironmental adaptation (see Text S1) using R [62].

### Genetic change over generations (family selection)

Natural family selection can result in genetic changes over generations for heritable traits. To test whether natural selection affected differently offspring from different families in Calderona (there was no survivors in Coca, see Results), we used a fractional paternity approach (similar to Devlin et al. [63]). Mother trees with incomplete genotypes were removed from the analyses (one mother from Calderona and six from Coca). Missing mothers may have reduced the power of the test, but fractional paternity approaches perform well with moderate numbers of unsampled potential parents [63], [64]. They also perform better than direct parent assignment when many ties are expected in parent-offspring links. First, LOD scores for each mother-offspring relationship, i.e. the probability to assign survivor offspring to candidate mothers based only on genotypes, were calculated using, respectively, three highly-discriminant nuSSRs for Coca mothers and four for Calderona's (see Materials and Methods). Second, LOD scores were summed up across all offspring for each mother and divided by the sum of LOD scores across all mothers, to produce a relative measure of female reproductive success for each mother tree. This step was done, respectively, for 32 and 24 survivors from Coca and Calderona *origin*. Third, we tested for over-representation of specific families among surviving offspring by using a *G*-test to compare observed with expected (i.e. equal) female reproductive success. Unfortunately, low overall offspring survival (see Results) did not allow more detailed quantitative genetics analyses.

## Results

### Temporal patterns of emergence and early survival

Emergence was generally low, ∼2.73%, irrespectively of population origin. Total number of emergences (both experimental sites combined) was 985 (2.46%) and 1,203 (3.01%) seedlings from Coca and Calderona origins, respectively. Early survival was also very low. Only 32 (3.25%) and 24 (1.99%) seedlings from Coca and Calderona origins, respectively, remained at the end of the experiment, all of them found in the milder Calderona site.

**Figure 2:**
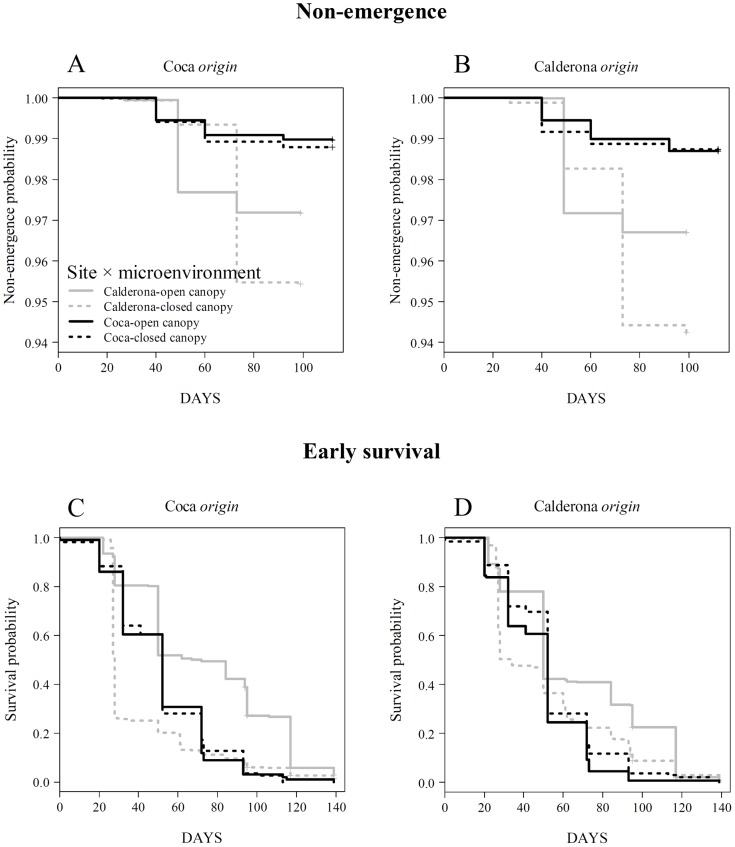
Kaplan-Meier estimators for non-emergence (A–B) and early survival (C–D) probability in the reciprocal sowing experiment. Four functions are shown, each corresponding to a combination of site and microenvironment (i.e. open- or closed-canopy cover, see legend in the figure). Differences across sites-microenvironments were highly significant (*p*<0.001), as shown by log-rank tests (see Table S1).

Emergence temporal patterns were very similar for both origins in the two experimental sites (Figure 2, top; here displayed as the probability of a seed of staying in the ground, i.e. the ‘non-emergence’), although there were significant differences (*p*<0.001, Table S1) across sites and local environments along the temporal axis (higher overall emergence in Calderona and under closed canopy). Around 40 days after seeds were sown, there was a big pulse of seedling emergences in Calderona, specifically under open canopy, but not in Coca. Moreover, around day 70 a shift in the suitable local environment to emerge took place, from open-canopy to closed-canopy areas, although, once again, only in Calderona. With respect to early survival, the two populations assayed showed a different temporal pattern (Figure 2, bottom). Calderona *origin* did not show marked differences across sites and canopy cover (i.e. low plasticity) while survival curves along time for Coca *origin* ran more distanced among them, evidencing a higher survival under open canopy in Calderona and a lower one under closed canopy in this same site. Nevertheless, in both cases log-rank tests were significant for both origins (*p*<0.001, Table S1).

### Local adaptation and the role of the microenvironment

Discrete-time logistic models were fitted to unveil ecological and genetic factors related to emergence and early survival in maritime pine, in particular in the context of local adaptation. The emergence process was driven by the following highly-significant factors (*p*<0.001): study site, population origin, microenvironment (i.e. the canopy-cover class) and the interaction term of site × microenvironment (Table 1). Neither the interaction term of site × population's origin (the standard test of local adaptation) nor the local environment × population's origin one were statistically significant. The significant interaction indicated a different effect of microenvironment across sites: Coca *site* had an overall lower emergence risk than Calderona *site* but it was lower under closed canopy (0.23) than in open canopy (0.36), despite the overall lower emergence risk under open canopy (0.59). Seedlings from Coca *origin* had a lower emergence risk (0.81) than Calderona *origin*, the reference level. Finally, considering emergence time intervals, the probability to emerge was higher between the second and third field census, which corresponded to about between 22 and 85 days after seeds were sown (Table 1).

**Table 1:** Odds ratios (OR) for the discrete-time logistic emergence model.

Factor	OR	SE	z	*P*>z	95% CI	
Site (Calderona)						
Coca	0.23	0.02	−17.24	0.00	0.19	0.27
Origin (Calderona)						
Coca	0.81	0.05	−3.57	0.00	0.72	0.82
Microenvironment (Closed canopy)						
Open canopy	0.59	0.04	−8.11	0.00	0.52	0.67
Site × origin	1.07	0.11	0.66	0.51	0.87	1.32
Site × microenvironment	1.59	0.17	4.38	0.00	1.29	1.95
Origin × microenvironment	1.01	0.09	0.16	0.87	0.85	1.21
Time intervals						
First	0.03	0.01	−18.20	0.00	0.02	0.04
Third	1.06	0.05	1.39	0.17	0.97	1.16
Fourth	0.09	0.01	−21.47	0.00	0.08	0.12

Number of observations generated: 316,935 (80,000 seeds)

Calderona *site*, Calderona *origin*, closed canopy, and the second-time interval (between census one and two, c. 20-50 days after sowing) are the reference levels (given also between parentheses for each factor/model). SE: standard error; CI: confidence intervals.

**Figure 3:**
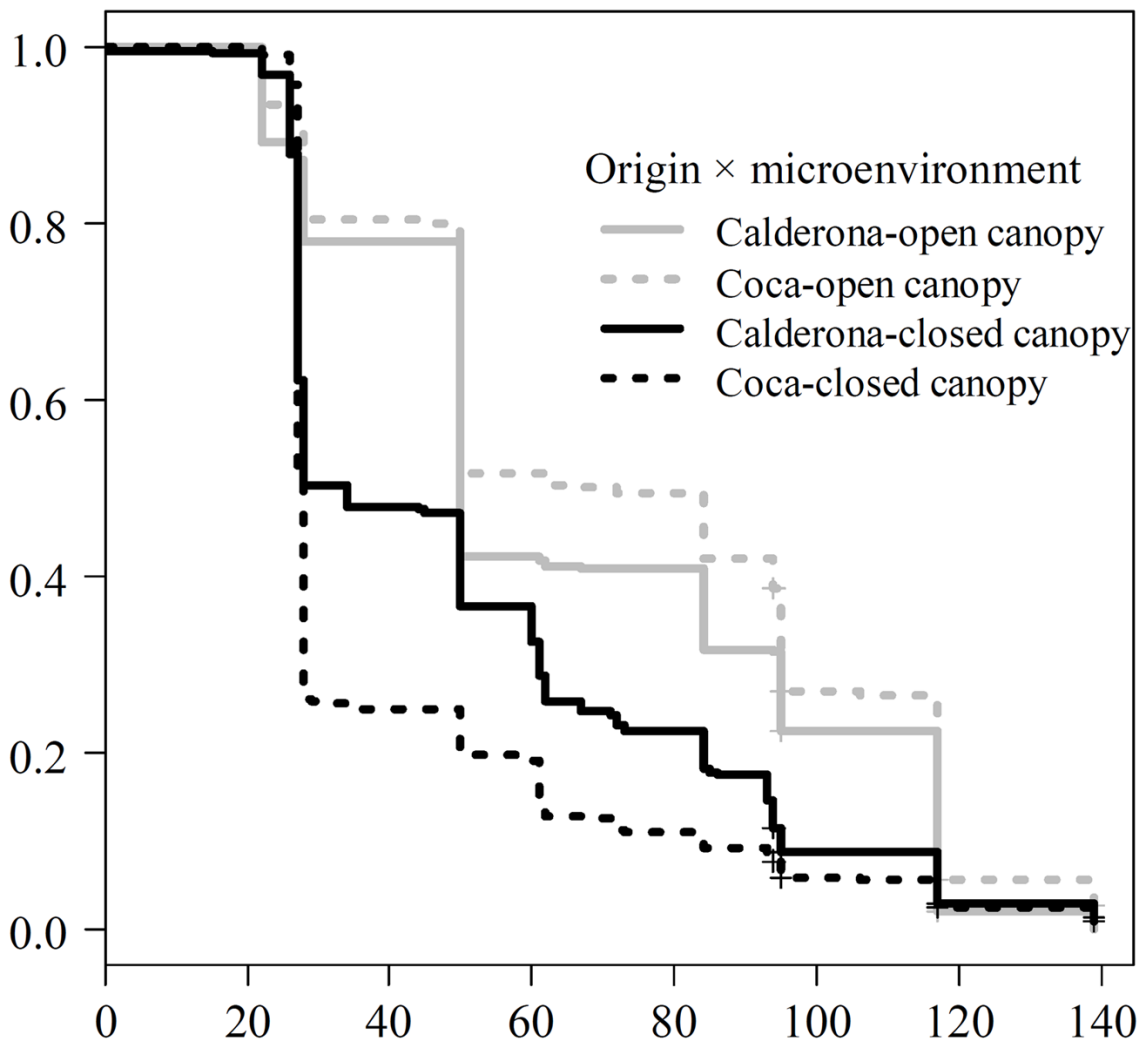
Kaplan-Meier estimators for early survival in Calderona, for two geographically-distant maritime pine origins. Four functions are shown, each one corresponding to a combination of origin and microenvironment (i.e. open- or closed-canopy cover, see legend in the figure). Differences across origins-microenvironments were highly significant (*p*<0.001), as shown by log-rank tests (see Table S1).

Emergence time, study site, population origin and the two interaction terms of population's origin × microenvironment and site × microenvironment were highly significant (*p*<0.01) in early survival models, while the interaction term of population's origin × site, i.e. the standard test of local adaptation, was not significant (*p* = 0.94) (Table 2). Date of emergence played an important role for early survival, with each additional day that a seed had taken to emerge increasing c. 8% death probability. Considered together, the interaction terms pointed to a lack of local adaptation but a high effect of microenvironment. Overall, the best environment for survival was Calderona *site* (Calderona was the only site with survivors) under open canopy (Table 2). Survival of Coca *origin* seedlings (i.e. the foreign population) was lower (OR of 0.62) than Calderona's (i.e. the local population) but only under closed canopy, Coca *origin* performing better under open canopy (OR of 1.26; see also Figure 3 and Table S1). Finally, probability of surviving was similar all along the experiment, except for an accused increase of mortality towards its end (between days 93 and 117 after sowing).

**Table 2:** Odds ratios (OR) for the discrete-time logistic survival model.

Factor	OR	SE	z	*P*>z	95% CI	
Time to emergence	0.93	0.00	21.18	0.00	0.93	0.94
Site (Calderona)						
Coca	0.45	0.31	5.54	0.00	0.34	0.60
Origin (Calderona)						
Coca	0.62	0.16	4.81	0.00	0.51	0.75
Microenvironment (Closed canopy)						
Open canopy	1.09	0.09	−0.83	0.41	0.89	1.33
Site × origin	1.01	0.16	−0.08	0.94	0.74	1.39
Site × microenvironment	0.63	0.27	2.78	0.01	0.45	0.87
Origin × microenvironment	2.04	0.07	−5.29	0.00	1.56	2.70
Time intervals						
First	2.33	0.04	−9.95	0.00	2.00	2.78
Third	2.00	0.06	−6.24	0.00	1.61	2.50
Fourth	1.16	0.11	−1.23	0.22	0.92	1.49
Fifth	0.12	1.58	11.64	0.00	0.08	0.17

Number of observations generated: 4,560 (2,188 seedlings)

Calderona *site*, Calderona *origin*, closed canopy, and the second-time interval (between census one and two, c. 20–52 days after seedlings emerged) are the reference levels (given also between parentheses for each factor/model). SE: standard error; CI: confidence intervals.

Analyses based on Hedges' g* effect sizes for both germination and survival showed similar results to discrete-time logistic models (see Text S1).

### Genetic change over generations (family selection)

There were only 56 survivors at the end of the experiment and all of them were found in milder-climate Calderona *site*. Fractional paternity analyses showed relative mother reproductive success from 0.054 to 4.195 for Coca's mothers and from 0.231 to 2.035 for Calderona's (Table S2). *G*-tests testing the hypothesis of equal effective number of offspring across mothers showed that survivors in this site were a random representation of the families included in the reciprocal sowing experiment (*G*-test = 33.659, d.f. = 42, *p*-value = 0.817), with selection not specifically favoring any family. However, significant differences were found when only mother-trees from Coca's origin were considered (*G*-test = 29.074, d.f. = 18, *p*-value = 0.047).

## Discussion

Successful recruitment was low in Mediterranean maritime pine, with just a few survivors in the milder Calderona *site*. This high level of mortality during the first growing season has often been reported in previous studies [40], [65]. Using a semi-natural reciprocal sowing experiment and discrete-time logistic models, we found that Calderona *origin* outperformed Coca *origin* in both emergence and survival, regardless of the study site. Thus, despite of the moderate-high level of genetic diversity reported for maritime pine in the Iberian Peninsula and high selection pressure at early stages of establishment, we did not find evidence supporting locally adapted populations at this stage. Furthermore, microenvironment heterogeneity (as exemplified here by canopy cover, a relevant ecological factor affecting soil microhabitat and radiation intensity) played a key role driving emergence and early survival differences when site conditions were not too harsh (i.e. in the milder Calderona *site*). Thus, some microenvironments within populations may favor foreign origins that otherwise would perform worse than local ones. Finally, we identified time of emergence as a key factor for seedling survival in stressful Mediterranean environments.

### Optimal environment for maritime pine early recruitment

Water availability is the major limiting factor for maritime pine recruitment [36]. We found an overall better performance in both emergence and early survival in Calderona *site*. These results clearly reflected the harsher (warmer and drier) climatic conditions in Coca *site* compared to those in Calderona *site*. Apart from the climatic conditions during recruitment, the spring and autumn precipitation from the previous year also affect natural regeneration success in this species [36]. Calderona *site* had c. 1.46 times more precipitation the year before and during our experiment than Coca *site*. Moreover, sandy soils in Coca further limit water availability, are a poor source of nutrients, and can result in extreme soil temperatures (as high as 60 °C) [66]. The importance of soil surface and horizon characteristics, and thus soil water and nutrient availability, has been highlighted for pine recruitment [46], [67]. Extreme environmental conditions in Coca would also explain the relative low importance of microenvironment (evaluated as open- or closed- canopy cover) in this site. In contrast, less stressful conditions for seedlings in Calderona *site* allowed microenvironment to play a key role for emergence and early survival processes. This is in agreement with previous research in Mediterranean plants [2].

### Effect of time of emergence in early survival

Date of emergence played an important role in early seedling survival, with a daily decrease in the probability of survival of c. 8%. This result is in agreement with previous studies in different environments and plants that found that seedlings that emerge earlier have also higher survival rates (e.g. in annual species in an early old-field plant community [68], in a temperate deciduous forest with *Fagus crenata* [69], and in Mediterranean forests with *Pinus sylvestris* [70]). Early emergence gives advantages for plant success in future life-history stages in different fitness components (survival, growth and/or fecundity) [71], [72]. For example, plants may develop a better root system, better competitive hierarchies or better access to light. Especially, in Mediterranean and arid environments, an early emergence enables seedlings to grow sufficiently during spring –benefiting from better environmental conditions (e.g. in terms of soil moisture, temperature and water regime)– so as to go on to survive the summer drought [70]. However, the critical period in the establishment phase takes place when the seedling changes its dependence from cotyledon reserves to its own photosynthesis [73]. Short, stressful events at this time, such as a few days with extreme temperatures, can be critical. In our study, the benefits of early emergence seemed to overcome the additional mortality risks entailed by Mediterranean climate heterogeneity.

### Lack of local adaptation at early recruitment stages

Considering a strict definition of local adaptation [29], we did not find evidence for local adaptation at early stages of establishment in the two population assayed, as we did not detect an overall better performance of local populations with respect to foreign ones using two complementary approaches (discrete-time logistic models and Hedges' g* effect sizes). In fact, Calderona *origin* outperformed the local origin in Coca at the emergence stage whereas microenvironment was more relevant than population's origin for survival performance in Calderona *site* (the only one with survivors).

In contrast with our study, local adaptation has often been reported for contrasted plant populations (see examples in reviews by Leimu and Fischer [31] and Hereford [32]; but notice that publication bias is expected to be large). Evidence for local adaptation in forest trees is also growing, thanks in part to the existence of large common gardens, in which more precise phenotypic evaluation is possible in comparison with semi-natural experiments. In general, these studies reflected a correlation of phenotypic variation with environmental factors (reviewed in [22], [74]). However, evidence of local adaptation in forest trees is more limited when strict tests associated to reciprocal transplants experiments are used. In long-lived species, such as forest trees, selection pressure can vary along life stages, which would blur local adaptation patterns measured at any given life stage. In addition, local adaptation in the populations studied may be associated with adult traits not expressed in seedlings (e.g. those related to adaptation to wildfire). Finally, given the high climatic heterogeneity across years of Mediterranean environments, our results may not be representative of the long-term evolution of the population, with years of better recruitment probably having more importance than those with low seedling survival (as the one studied).

The lack of local adaptation in our study could also be related to the traits measured. Hereford's meta-analysis [32] found a greater magnitude of local adaptation when fecundity or composite measures of fitness (i.e. measures combining viability, usually survival and fecundity) were computed instead of just raw survival measurements. It was also suggested that a measure of viability, such as survival, might have less variance than fecundity or other fitness components, which may result into less accurate estimates of selection and local adaptation [75]. This would be exacerbated by the typically low power in semi-natural experiments (due to lesser environmental control). Another possible explanation could be related to population-specific recruitment strategies that confer higher overall fitness to some populations. For example, emergence outperformance by Calderona *origin* (1.23 times that of Coca) could be related to adaptation to high fire recurrence, which might have selected faster emergence relative to other populations within the species. In contrast, the greater climatic heterogeneity and sparse tree density in Coca might have selected for more moderate rates of emergence in this site.

### Role of microenvironmental (canopy cover) variation

Microenvironment affected seedling's performance and modulated local adaptation patterns. In particular, Calderona's *origin* outperformed Coca's for survival at its home site but only under closed canopy. These results highlight two fundamental aspects affecting recruitment success in forest trees. On the one hand, they reflect variation in fitness-related performance traits due to specific microenvironment properties. Previous studies in *Pinus pinaster* have shown that successful recruitment depends on local climate and stand characteristics [40], [65]. In American oaks (*Quercus rubra*), Sork et al. [76] found local adaptation to herbivory in five year old seedlings from adjacent populations. On the other hand, better performance in particular microenvironments could be associated to population-specific life-history strategies. For example, Coca population, originated in the harsh environment of the Castilian central plateau, could present a life-history strategy better adapted to drier environments, which could have promoted its better performance under open canopy in milder sites. Drought-tolerant maritime pine populations have higher root biomass allocation [77], [78] and this seems a plausible mechanism for Coca *origin*'s better performance than local origins in open-canopy microenvironment. However, this hypothesis remains untested and new experiments would be needed to elucidate population-specific adaptive strategies. Overall, our results suggest the necessity to include microenvironment variation within populations, as well as climate variation across years, to test for local adaptation, in particular in systems where environment (and, consequently, selection pressure) may vary at the fine temporal and spatial scales.

Expected shifts from open to closed microenvironments were observed during the emergence stage but not during the early survival stage. A plausible explanation for the latter could be that, as the growing season progresses, closed-canopy areas develop more suitable conditions to emerge, e.g. higher soil water retention. Greater emergence under canopy cover has been reported for maritime pine in previous studies [40], [79]. Nevertheless, these differences tend to disappear with time due to widespread mortality.

### Family selection and potential for genetic change

The differential action of natural selection at the family level would, in principle, allow for microevolutionary change, given heritable adaptive traits [80] (i.e. traits that respond to selection). In our study, some families of Coca *origin* produced a higher number of offspring than expected, assuming an equal survival in Calderona (the only site with survivors). These results suggest that Coca *origin* might contain more genetic variance available for selection than the local Calderona *origin*. However, apart from family selection, other factors, such as differences in seed quality among families, may have caused these differences. Moreover, our test combines absence of germination and seedling mortality, and thus family differences respond to multiple, not exclusive biological processes. Lack of family differences in the local (Calderona) origin could result from reduced genetic variance due to demographical history, past episodes of local adaptation, and/or absence of relevant microevolution drivers at early stages of establishment (e.g. wildfire). More detailed quantitative genetic field experiments including multiple populations would be needed to further evaluate genetic responses of contrasted maritime pine origins in the wild.

### Conclusions

In this study under semi-natural conditions, we did not find evidence for local adaptation in Mediterranean maritime pine at the early stages of its life cycle, with one of the studied origins outperforming the other in both sites. Apart from population origin, our study suggests that under stressful conditions with low survival, stochastic factors could be more important than natural selection at early stages of recruitment in forest trees. Moreover, if confirmed at adult stages, our study provides information to develop mitigation strategies in the face of climate change. For example, for assisted migration applications, i.e. the transplantation of populations following climate change, our results suggest that naturally pre-adapted foreign populations (or those containing higher levels of genetic variance) may perform better than local ones. However, we have shown that this process is modulated by microenvironmental variation, and that origin selection needs to account not only for climatic suitability, but also for population life-history strategies and the high stochasticity found for recruitment in stressful Mediterranean environments.

## Supporting Information

Figure S1 Soil temperature in Calderona and Coca experimental *sites*. Data recorded with a HOBO data logger from May to September.
(PDF)

Figure S2 Mean, maximum and minimum air temperature (°C), and monthly precipitation (mm) in Calderona and Coca experimental sites. Red circles represent climate data recorded over the study year and box-plots the historical data between 1983 and 2013 from the closest meteorological stations with long climate series (Olmedo, c. 17 km from Coca, and Beteta, c. 13 km from Calderona) corrected using Gonzalo's phitoclimatic model for Spain [44].
(PDF)

Figure S3 Pictures showing initial soil conditions in the two experimental sites. Top, Coca *site*, where small shrubs are rare. Bottom, Calderona *site*; on the left side, an experimental plot is under preparation by removing small shrubs using pruning-scissors; the right side shows initial soil conditions.
(PDF)

Figure S4 Pictures showing our semi-natural sowing reciprocal experiment. The bigger picture presents a general overview, centered in one experimental plot. This picture is complemented with four insets in its corners. Each inset shows different growing conditions for seedlings. On the left side, Coca *site*; and on the right, Calderona *site*.
(PDF)

Table S1 Log-rank tests to assess heterogeneous patterns in Kaplan-Meier (KM) functions for non-emergence and early survival probability: (i) site-microenvironment combinations for each origin, Coca or Calderona (see Figure 2); and (ii) origin-microenvironment combinations in Calderona site (only for survival, see Figure 3).
(PDF)

Table S2 Female reproductive success measures for each mother tree. a) Mother trees from Calderona *origin*. b) Mother trees from Coca *origin*. NA: Missing data.
(PDF)

Text S1 Effect sizes (Hedges' g*) for local and microenvironmental adaptation.
(PDF)

Data S1 Raw data.
(XLS)

## References

[1] Pérez-Ramos IM, Rodríguez-Calcerrada J, Ourcival JM, Rambal S (2013) *Quercus ilex* recruitment in a drier world: a multi-stage demographic approach. Perspect Plant Ecol Evol Syst 15: 106–117 10.1016/j.ppees.2012.12.005

[2] Gómez-Aparicio L (2008) Spatial patterns of recruitment in Mediterranean plant species: linking the fate of seeds, seedlings and saplings in heterogeneous landscapes at different scales. J Ecol 96: 1128–1140 10.1111/j.1365-2745.2008.01431.x

[3] Clark JS, Beckage B, Camill P, Cleveland B, Hillerislambers J, et al (1999) Interpreting recruitment limitation in forests. Am J Bot 86: 1–16. 21680341

[4] Muller-Landau HC, Wright SJ, Calderón O, Hubbell SP, Foster RB (2002) Assessing recruitment limitation: concepts, methods and case-studies from a tropical forest. In: Levey DJ, Silva WR, Galetti M, editors. Seed dispersal and frugivory: ecology, evolution and conservation. CAB International. pp. 35–53.

[5] Facelli JM, Pickett STA (1991) Plant litter: its dynamics and effects on plant community structure. Bot Rev 57: 1–32 10.1007/BF02858763

[6] Kobe RK, Pacala SW, Silander JA, Canham CD (1995) Juvenile tree survivorship as a component of shade tolerance. Ecol Appl 5: 517–532 10.2307/1942040

[7] Augspurger CK (1984) Seedling survival of tropical tree species: interactions of dispersal distance, light-gaps, and pathogens. Ecology 65: 1705–1712 10.2307/1937766

[8] Pigott CD, Pigott S (1993) Water as a determinant of the distribution of trees at the boundary of the Mediterranean zone. J Ecol 81: 557–566.

[9] Castro J, Zamora R, Hódar JA, Gómez JM (2005) Alleviation of summer drought boosts establishment success of *Pinus sylvestris* in a Mediterranean mountain: an experimental approach. Plant Ecol 181: 191–202 10.1007/s11258-005-6626-5

[10] Aranda I, Alía R, Ortega U, Dantas ÂK, Majada J (2009) Intra-specific variability in biomass partitioning and carbon isotopic discrimination under moderate drought stress in seedlings from four *Pinus pinaster* populations. Tree Genet Genomes 6: 169–178 10.1007/s11295-009-0238-5

[11] Voltas J, Chambel MR, Prada MA, Ferrio JP (2008) Climate-related variability in carbon and oxygen stable isotopes among populations of Aleppo pine grown in common-garden tests. Trees 22: 759–769 10.1007/s00468-008-0236-5

[12] Sánchez-Gómez D, Robson TM, Gascó A, Gil-Pelegrín E, Aranda I (2013) Differences in the leaf functional traits of six beech (*Fagus sylvatica* L.) populations are reflected in their response to water limitation. Environ Exp Bot 87: 110–119 10.1016/j.envexpbot.2012.09.011

[13] Klein T, Di Matteo G, Rotenberg E, Cohen S, Yakir D (2013) Differential ecophysiological response of a major Mediterranean pine species across a climatic gradient. Tree Physiol 33: 26–36 10.1093/treephys/tps116 23192974

[14] Harper J (1977) Population biology of plants. New York: Academic Press.

[15] Herrera CM, Jordano P, Lopez-Soria L, Amat JA (1994) Recruitment of a mast-fruiting, bird-dispersed tree: bridging frugivore activity and seedling establishment. Ecol Monogr 64: 315–344 10.2307/2937165

[16] Rodríguez-García E, Ordóñez C, Bravo F (2011) Effects of shrub and canopy cover on the relative growth rate of *Pinus pinaster* Ait. seedlings of different sizes. Ann For Sci 68: 337–346 10.1007/s13595-011-0039-5

[17] Peet RK, Christensen NL (1987) Competition and tree death. Bioscience 7: 586–595.

[18] Donohue K, Rubio de Casas R, Burghardt L, Kovach K, Willis CG (2010) Germination, postgermination adaptation, and species ecological ranges. Annu Rev Ecol Evol Syst 41: 293–319 10.1146/annurev-ecolsys-102209-144715

[19] Reich PB, Wright IJ, Craine JM, Oleksyn J, Westoby M, et al (2003) The evolution of plant functional variation: traits, spectra, and strategies. Int J Plant Sci 164: 143–164.

[20] Franks SJ, Sim S, Weis AE (2007) Rapid evolution of flowering time by an annual plant in response to a climate fluctuation. Proc Natl Acad Sci USA 104: 1278–1282 10.1073/pnas.0608379104 17220273 PMC1783115

[21] Davis M, Shaw R (2001) Range shifts and adaptive responses to Quaternary climate change. Science 292: 673–679. 11326089 10.1126/science.292.5517.673

[22] Savolainen O, Pyhäjärvi T, Knürr T (2007) Gene flow and local adaptation in trees. Annu Rev Ecol Evol Syst 38: 595–619 10.1146/annurev.ecolsys.38.091206.095646

[23] Endler J (1977) Geographic variation, speciation and clines. Princeton, New Jersey: Princeton University Press. 409931

[24] Barton NH (1999) Clines in polygenic traits. Genet Res 74: 223–236. 10689800 10.1017/s001667239900422x

[25] Aitken SN, Yeaman S, Holliday JA, Wang T, Curtis-McLane S (2008) Adaptation, migration or extirpation: climate change outcomes for tree populations. Evol Appl 1: 95–111 10.1111/j.1752-4571.2007.00013.x 25567494 PMC3352395

[26] Klenner W, Arsenault A (2009) Ponderosa pine mortality during a severe bark beetle (Coleoptera: *Curculionidae*, *Scolytinae*) outbreak in southern British Columbia and implications for wildlife habitat management. For Ecol Manage 258: S5–S14 10.1016/j.foreco.2009.08.035

[27] Rehfeldt GE, Ying CC, Spittlehouse DL, Hamilton DA (1999) Genetic responses to climate in *Pinus contorta*: Niche breadth, climate change, and reforestation. Ecol Monogr 69: 375–407.

[28] Savolainen O, Bokma F, García-Gil R, Komulainen P, Repo T (2004) Genetic variation in cessation of growth and frost hardiness and consequences for adaptation of *Pinus sylvestris* to climatic changes. For Ecol Manage 197: 79–89 10.1016/j.foreco.2004.05.006

[29] Kawecki TJ, Ebert D (2004) Conceptual issues in local adaptation. Ecol Lett 7: 1225–1241 10.1111/j.1461-0248.2004.00684.x

[30] Primack RB, Kang H (1989) Measuring fitness and natural selection in wild plant populations. Ann Rev Ecol Syst 20: 367–396.

[31] Leimu R, Fischer M (2008) A meta-analysis of local adaptation in plants. PLoS One 3: e4010 10.1371/journal.pone.0004010 19104660 PMC2602971

[32] Hereford J (2009) A quantitative survey of local adaptation and fitness trade-offs. Am Nat 173: 579–588 10.1086/597611 19272016

[33] Schupp EW (1995) Seed-seedling conflicts, habitat choice, and patterns of plant recruitment. Am J Bot 82: 399–409 10.2307/2445586

[34] Chambers JC (2001) *Pinus monophylla* establishment in an expanding Pinus-Juniperus woodland: environmental conditions, facilitation and interacting factors. J Veg Sci 12: 27 10.2307/3236671

[35] Kollmann J (2000) Dispersal of fleshy-fruited species: a matter of spatial scale? Perspect Plant Ecol Evol Syst 3: 29–51 10.1078/1433-8319-00003

[36] Rodriguez-Garcia E, Gratzer G, Bravo F (2011) Climatic variability and other site factor influences on natural regeneration of *Pinus pinaster* Ait. in Mediterranean forests. Ann For Sci 68: 811–823 10.1007/s13595-011-0078-y

[37] Gómez JM (2004) Importance of microhabitat and acorn burial on *Quercus ilex* early recruitment: non-additive effects on multiple demographic processes. Plant Ecol 172: 287–297 10.1023/B:VEGE.0000026327.60991.f9

[38] González-Martínez SC, Alía R, Gil L (2002) Population genetic structure in a Mediterranean pine (*Pinus pinaster* Ait.): a comparison of allozyme markers and quantitative traits. Heredity 89: 199–206 10.1038/sj.hdy.6800114 12209390

[39] Verdú M, Pausas JG (2007) Fire drives phylogenetic clustering in Mediterranean basin woody plant communities. J Ecol 95: 1316–1323 10.1111/j.1365-2745.2007.01300.x

[40] Ruano I, Pando V, Bravo F (2009) How do light and water influence *Pinus pinaster* Ait. germination and early seedling development? For Ecol Manage 258: 2647–2653 10.1016/j.foreco.2009.09.027

[41] Tapias R, Pardos JA, Gil L, Climent J (2004) Life histories of Mediterranean pines. Plant Ecol 171: 53–68.

[42] Ishizuka W, Goto S (2012) Modeling intraspecific adaptation of *Abies sachalinensis* to local altitude and responses to global warming, based on a 36-year reciprocal transplant experiment. Evol Appl 5: 229–244 10.1111/j.1752-4571.2011.00216.x 25568044 PMC3353353

[43] Giménez-Benavides L, Escudero A, Iriondo JM (2007) Local adaptation enhances seedling recruitment along an altitudinal gradient in a high mountain mediterranean plant. Ann Bot 99: 723–734 10.1093/aob/mcm007 17307775 PMC2802927

[44] Gonzalo Jiménez J (2008) Diagnosis fitoclimática de la España peninsular. Actualización y análisis geoestadístico aplicado. PhD Dissertation, Universidad Politécnica de Madrid.

[45] Anderson MC (1964) Studies of the woodland light climate: I. The photographic computation of light conditions. J Ecol 52: 27–41 10.2307/2257780

[46] Juez L, González-Martínez SC, Nanos N, De-Lucas AI, Ordóñez C, et al (2014) Can seed production and restricted dispersal limit recruitment in *Pinus pinaster* Aiton from the Spanish Northern Plateau? For Ecol Manage 313: 329–339.

[47] González-Martínez SC, Gerber S, Cervera MT, Martínez-Zapater JM, Gil L, et al (2003) Selfing and sibship structure in a two-cohort stand of maritime pine (*Pinus pinaster* Ait.) using nuclear SRR markers. Ann For Sci 60: 115–121.

[48] Chagné D, Chaumeil P, Ramboer A, Collada C, Guevara A, et al (2004) Cross-species transferability and mapping of genomic and cDNA SSRs in pines. Theor Appl Genet 109: 1204–1214. 15448894 10.1007/s00122-004-1683-z

[49] McNair JN, Sunkara A, Frobish D (2012) How to analyse seed germination data using statistical time-to-event analysis: non-parametric and semi-parametric methods. Seed Sci Res 22: 77–95 10.1017/S0960258511000547

[50] Kaplan EL, Meier P (1958) Nonparametric estimation from incomplete observations. J Am Stat Assoc 53: 457–481.

[51] Onofri A, Carbonell EA, Piepho HP, Mortimer AM, Cousens RD (2010) Current statistical issues in weed research. Weed Res 50: 5–24.

[52] Fox G (2001) Failure–time analysis: emergence, floxwering, survivorship, and other waiting times. Design and analysis of ecological experiments. New York: Oxford University Press. pp. 235–266.

[53] Terry T (2012) A package for survival analysis in S. R package version 2.37-4.

[54] R Core Team (2012) R: A language and environment for statistical computing.

[55] Allison PD (1982) Discrete-time methods for the analysis of event histories. Sociol Methodol 13: 61–98.

[56] Kalbfleisch JD, Prentice RL (1980) The statistical analysis of failure time data. 10.1002/9781118032985

[57] Singer JD, Willett J (2003) Applied longitudinal data analysis: modeling change and event occurrence. New York: Oxford University Press.

[58] Hosmer DW, Lemeshow S (1989) Applied logistic regression. Willey, New York.

[59] StataCorp (2009) Stata Statistical Software: Release 11.

[60] Hedges LV (1981) Distribution theory for glass's estimator of effect size and related estimators. J Educ Behav Stat 6: 107–128.

[61] Cohen J (1988) Statistical power analysis for the behavioral sciences. Erlbaum L, editor Hillsdale, NJ: Lawrence Erlbaum Associates. doi:10.1234/12345678.

[62] AC Del Re (2012) A package to compute effect sizes. R Development Core Team.

[63] Devlin B, Roeder K, Ellstrand NC (1988) Fractional paternity assignment: theoretical development and comparison to other methods. Theor Appl Genet 76: 369–380. 24232200 10.1007/BF00265336

[64] Jones AG, Ardren WR (2003) Methods of parentage analysis in natural populations. Mol Ecol 12: 2511–2523 10.1046/j.1365-294X.2003.01928.x 12969458

[65] Rodríguez-García E, Juez L, Bravo F (2010) Environmental influences on post-harvest natural regeneration of *Pinus pinaster* Ait. in Mediterranean forest stands submitted to the seed-tree selection method. Eur J For Res 129: 1119–1128 10.1007/s10342-010-0399-7

[66] Gordo J, Pardos M, Bravo F, Montero G (2012) La regeneración natural de los pinares en los arenales de la Meseta Castellana. Valladolid: Universidad de Valladolid-INIA.

[67] Rojo A, Montero G, Orgeta C (1994) Natural regeneration in *Pinus sylvestris* L. Investig Agrar Sist y Recur For Fuera Ser 3: 107–125.

[68] Miller TE (1987) Effects of emergence time on survival and growth in an early old-field plant community. Oecologia 72: 272–278. 28311550 10.1007/BF00379278

[69] Abe M, Honda A, Hoshizaki K, Miguchi H (2007) Advantage of early seedling emergence in *Fagus crenata*: importance of cotyledon stage for predator escape and pathogen avoidance. Ecol Res 23: 681–688 10.1007/s11284-007-0428-2

[70] Castro J (2006) Short delay in timing of emergence determines establishment success in *Pinus sylvestris* across microhabitats. Ann Bot 98: 1233–1240 10.1093/aob/mcl208 17056614 PMC2803580

[71] Verdú M, Traveset A (2005) Early emergence enhances plant fitness: a phylogenetically controlled meta-analysis. Ecology 86: 1385–1394 10.1890/04-1647

[72] Ross MA, Harper JL (1972) Occupation of biological space during seedling establishment. J Ecol 60: 77–88 10.2307/2258041

[73] Harper JL, White J (1974) The demography of plants. Annu Rev Ecol Syst 5: 419–463 10.1146/annurev.es.05.110174.002223

[74] Alberto F, Aitken SN, Alía R, González-Martínez SC, Kremer A, et al (2013) Evolutionary response to climate change – evidence from tree populations. Glob Chang Biol: 1–47. 10.1111/gcb.12181 PMC3664019 23505261

[75] Kingsolver JG, Hoekstra HE, Hoekstra JM, Berrigan D, Vignieri SN, et al (2001) The strength of phenotypic selection in natural populations. Am Nat 157: 245–261 10.1086/319193 18707288

[76] Sork V, Kirk S, Hochwender C (1993) Evidence for local adaptation in closely adjacent subpopulations of northern red oak (*Quercus rubra* L.) expressed as resistance to leaf herbivores. Am J Bot 142: 928–936. 10.1086/285581 19425941

[77] Benito Garzón M, Alía R, Robson TM, Zavala MA (2011) Intra-specific variability and plasticity influence potential tree species distributions under climate change. Glob Ecol Biogeogr 20: 766–778 10.1111/j.1466-8238.2010.00646.x

[78] Joslin JD, Wolfe HM, Hanson PJ (2000) Effects of altered water regimes on forest root systems. New Phytol 147: 117–129 10.1046/j.1469-8137.2000.00692.x

[79] Rodríguez-García E, Bravo F, Spies TA (2011) Effects of overstorey canopy, plant–plant interactions and soil properties on Mediterranean maritime pine seedling dynamics. For Ecol Manage 262: 244–251 10.1016/j.foreco.2011.03.029

[80] Pommel B, Mouraux D, Cappellen O, Ledent JF (2002) Influence of delayed emergence and canopy skips on the growth and development of maize plants: a plant scale approach with CERES-Maize. Eur J Agron 16: 263–277 10.1016/S1161-0301(01)00130-7

