# Supplementary material for: The Role of Population Origin and Microenvironment in Seedling Emergence and Early Survival in Mediterranean Maritime Pine (Pinus pinaster Aiton)
Source: PLoS One. 2014 Oct 6;9(10):e109132. doi: 10.1371/journal.pone.0109132 (PMC4186868; doi:10.1371/journal.pone.0109132)
Supplement: Figure S1 — Soil temperature in Calderona and Coca experimental sites. Data recorded with a HOBO data logger from May to September. (PDF) [file pone.0109132.s001.pdf]

Supporting Figure S1

Supporting Tables and Figures

The role of population origin and microenvironment in seedling emergence and early survival in Mediterranean maritime pine (*Pinus pinaster* Aiton)

Natalia Vizcaíno-Palomar, Bárbara Revuelta-Eugercios, Miguel A. Zavala, Ricardo Alía, Santiago C. González-Martínez*

*To whom correspondence should be addressed. E-mail: santiago@inia.es

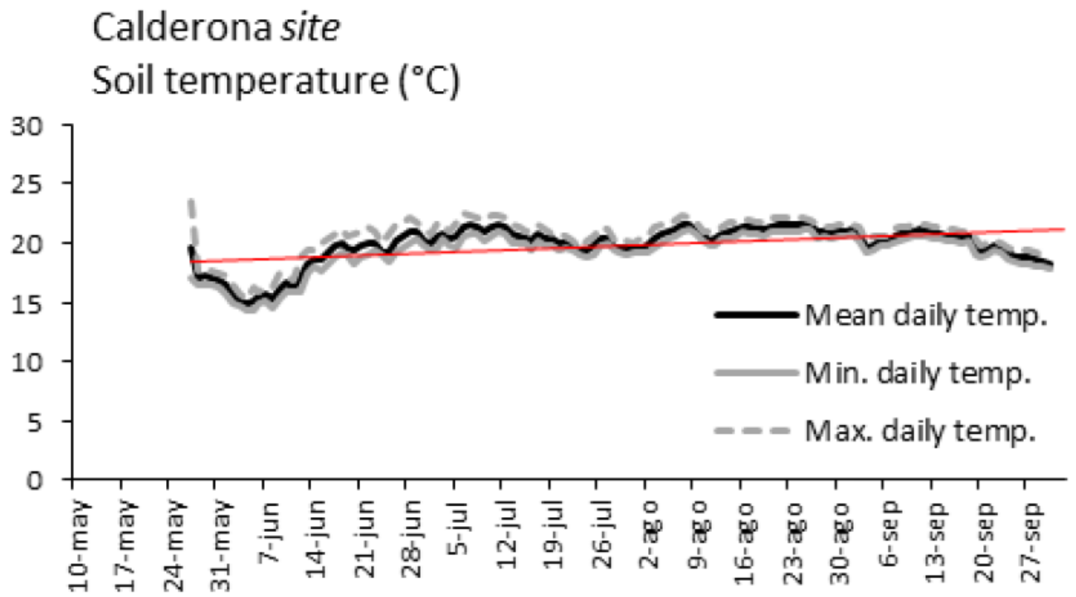

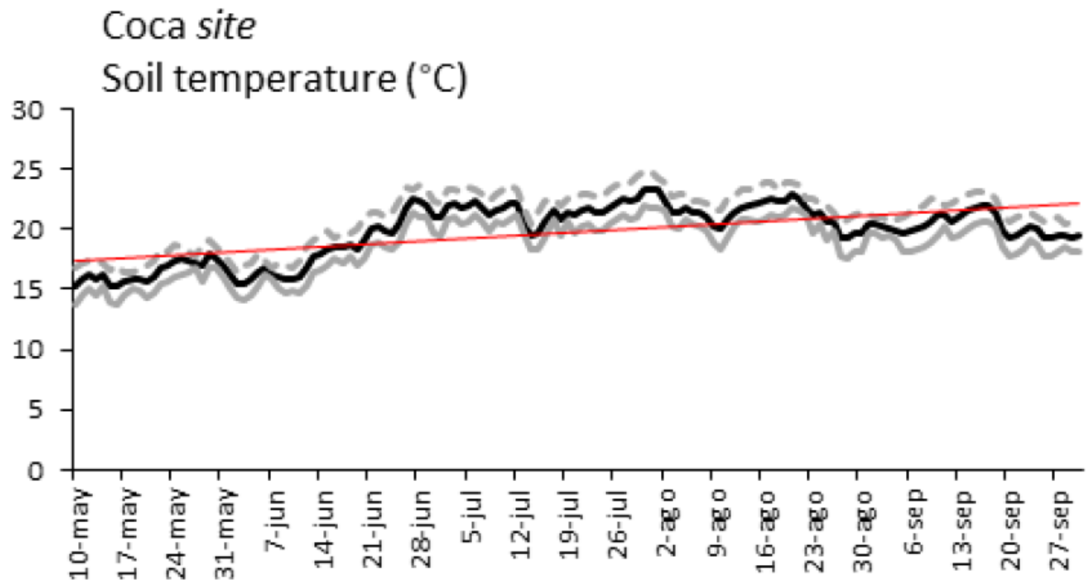

Figure S1. Soil temperature in Calderona and Coca experimental *sites*. Data recorded with a HOBO data logger from May to September.
