# Supplementary material for: The Role of Population Origin and Microenvironment in Seedling Emergence and Early Survival in Mediterranean Maritime Pine (Pinus pinaster Aiton)
Source: PLoS One. 2014 Oct 6;9(10):e109132. doi: 10.1371/journal.pone.0109132 (PMC4186868; doi:10.1371/journal.pone.0109132)
Supplement: Figure S2 — Mean, maximum and minimum air temperature (°C), and monthly precipitation (mm) in Calderona and Coca experimental sites. Red circles represent climate data recorded over the study year and box-plots the historical data between 1983 and 2013 from the closest meteorological stations with long climate series (Olmedo, c. 17 km from Coca, and Beteta, c. 13 km from Calderona) corrected using Gonzalo's phitoclimatic model for Spain [44]. (PDF) [file pone.0109132.s002.pdf]

Supporting Figure S2

Supporting Tables and Figures

The role of population origin and microenvironment in seedling emergence and early survival in Mediterranean maritime pine (*Pinus pinaster* Aiton)

Natalia Vizcaíno-Palomar, Bárbara Revuelta-Eugercios, Miguel A. Zavala, Ricardo Alía, Santiago C. González-Martínez*

*To whom correspondence should be addressed. E-mail: santiago@inia.es

Coca *site* **Calderona *site***

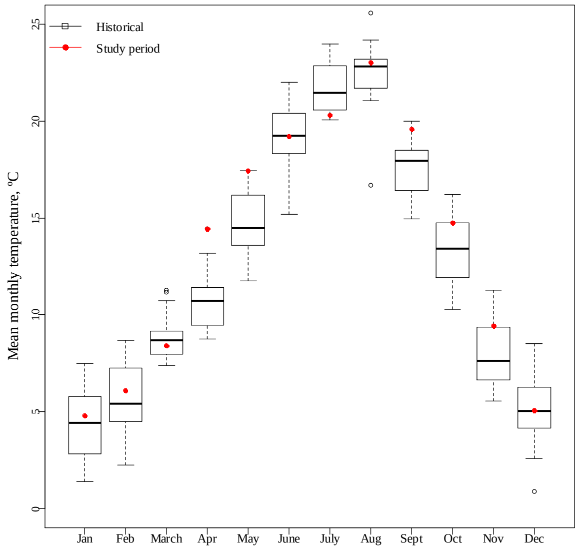

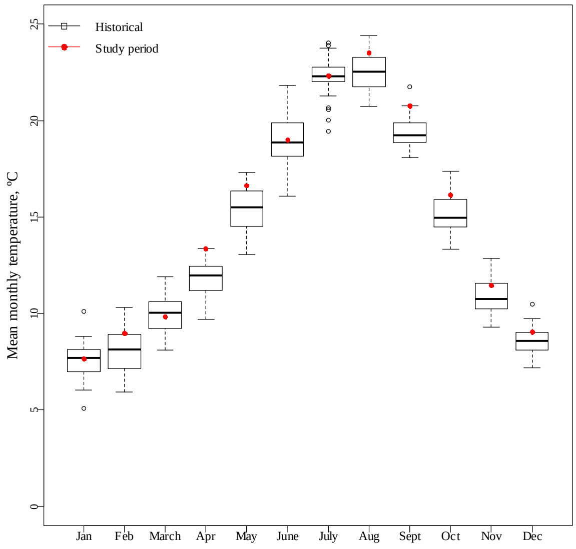

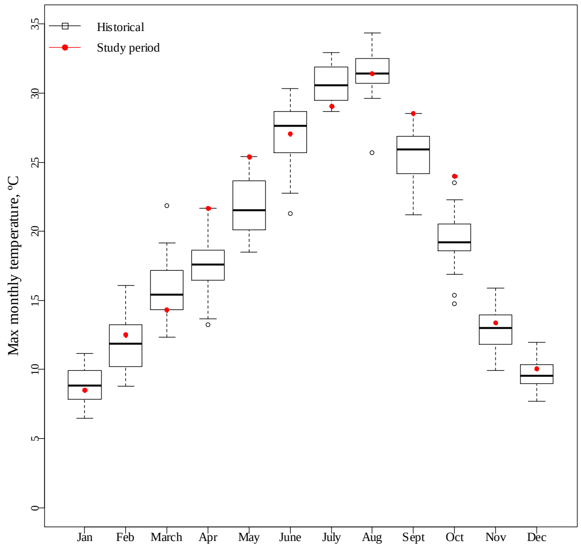

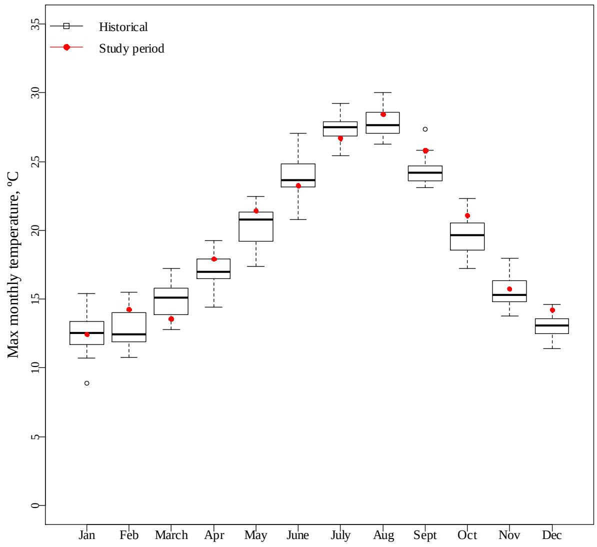

(continued in next page)

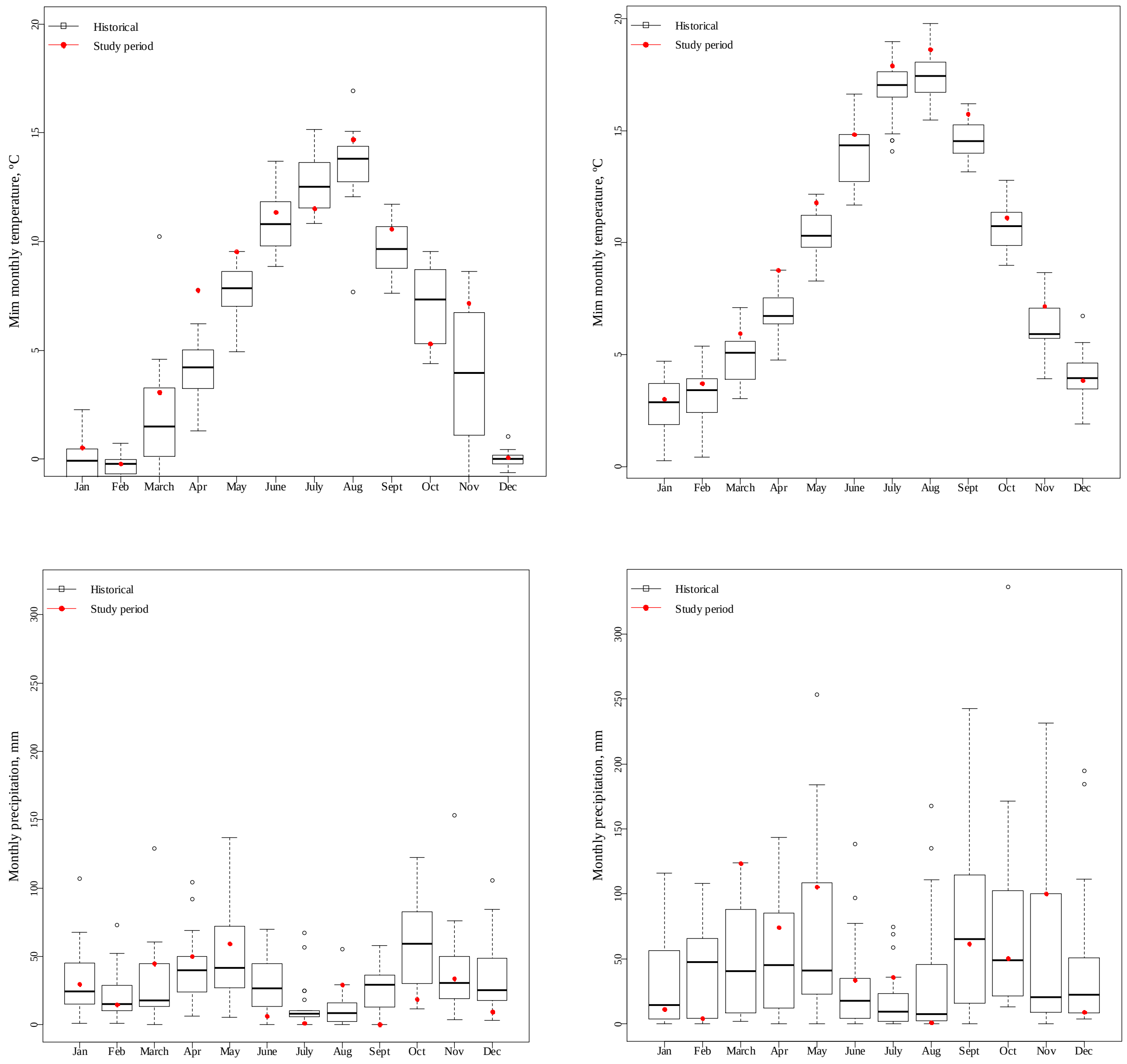

Figure S2. Mean, maximum and minimum air temperature (ºC), and monthly precipitation (mm) in Calderona and Coca experimental sites. Red circles represent climate data recorded over the study year and box-plots the historical data between 1983 and 2013 from the closest meteorological stations with long climate series (Olmedo, c. 17 km from Coca, and Beteta, c. 13 km from Calderona) corrected using Gonzalo's phitoclimatic model for Spain [44].
