# Supplementary material for: The Role of Population Origin and Microenvironment in Seedling Emergence and Early Survival in Mediterranean Maritime Pine (Pinus pinaster Aiton)
Source: PLoS One. 2014 Oct 6;9(10):e109132. doi: 10.1371/journal.pone.0109132 (PMC4186868; doi:10.1371/journal.pone.0109132)
Supplement: Figure S3 — Pictures showing initial soil conditions in the two experimental sites. Top, Coca site, where small shrubs are rare. Bottom, Calderona site; on the left side, an experimental plot is under preparation by removing small shrubs using pruning-scissors; the right side shows initial soil conditions. (PDF) [file pone.0109132.s003.pdf]

Supporting Figure S3

Supporting Tables and Figures

The role of population origin and microenvironment in seedling emergence and early survival in Mediterranean maritime pine (*Pinus pinaster* Aiton)

Natalia Vizcaíno-Palomar, Bárbara Revuelta-Eugercios, Miguel A. Zavala, Ricardo Alía, Santiago C. González-Martínez*

*To whom correspondence should be addressed. E-mail: santiago@inia.es

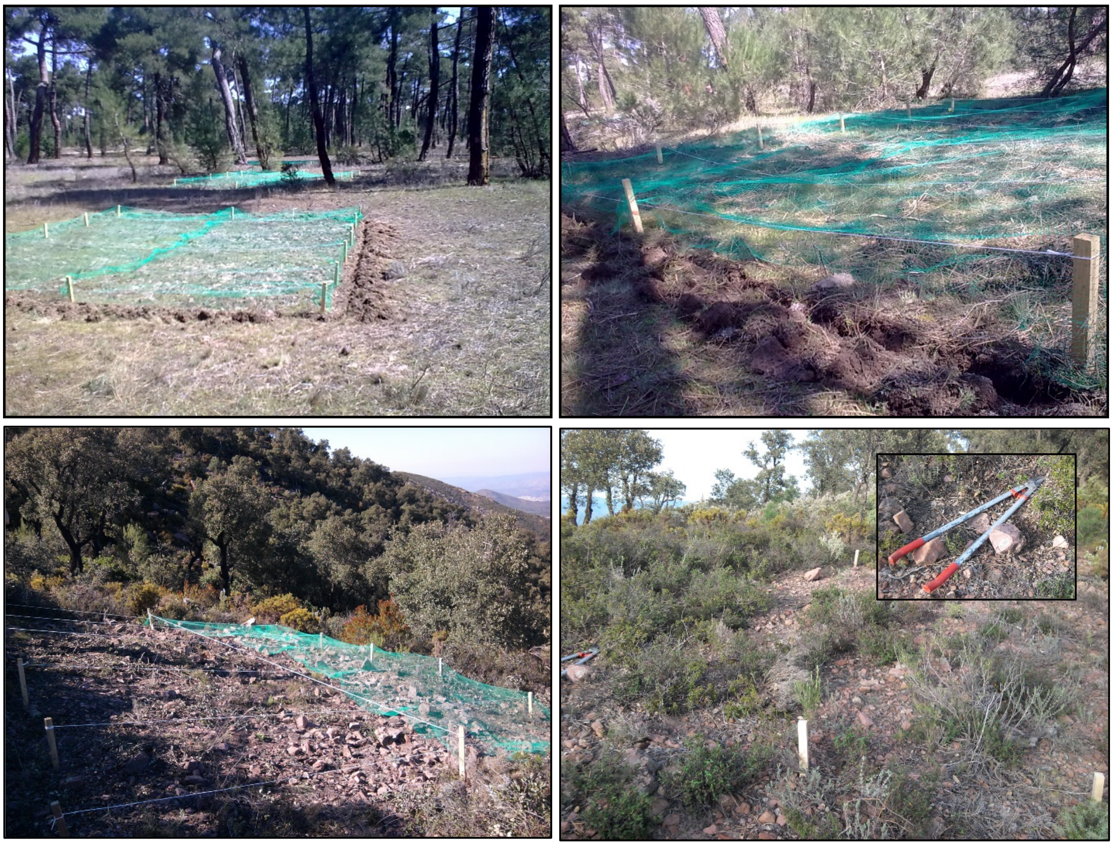

Figure S3. Pictures showing initial soil conditions in the two experimental sites. Top, Coca *site*, where small shrubs are rare. Bottom, Calderona *site*; on the left side, an experimental plot is under preparation by removing small shrubs using pruning-scissors; the right side shows initial soil conditions.
