# Supplementary material for: The Role of Population Origin and Microenvironment in Seedling Emergence and Early Survival in Mediterranean Maritime Pine (Pinus pinaster Aiton)
Source: PLoS One. 2014 Oct 6;9(10):e109132. doi: 10.1371/journal.pone.0109132 (PMC4186868; doi:10.1371/journal.pone.0109132)
Supplement: Figure S4 — Pictures showing our semi-natural sowing reciprocal experiment. The bigger picture presents a general overview, centered in one experimental plot. This picture is complemented with four insets in its corners. Each inset shows different growing conditions for seedlings. On the left side, Coca site; and on the right, Calderona site. (PDF) [file pone.0109132.s004.pdf]

# Supporting Figure S4

*Supporting Tables and Figures*

**The role of population origin and microenvironment in seedling emergence and early survival in Mediterranean maritime pine (*Pinus pinaster* Aiton)**

Natalia Vizcaíno-Palomar, Bárbara Revuelta-Eugercios, Miguel A. Zavala, Ricardo Alía, Santiago C. González-Martínez*

*To whom correspondence should be addressed. E-mail: santiago@inia.es

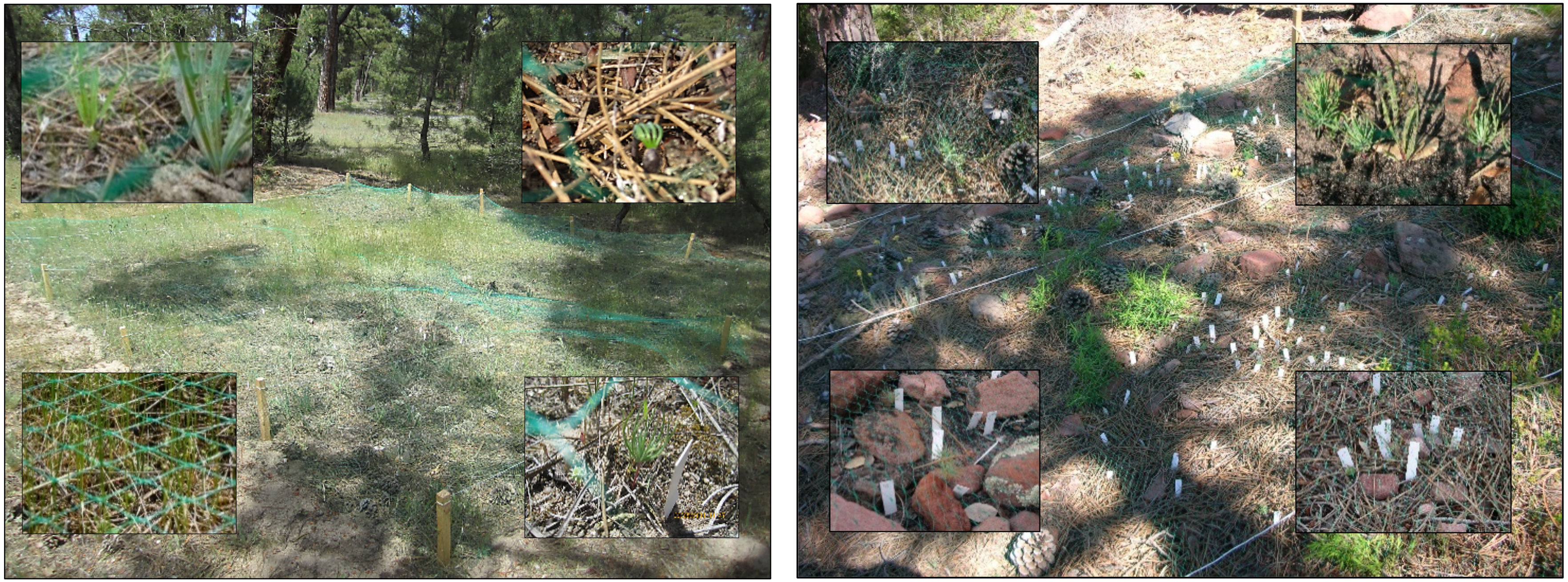

**Figure S4.** Pictures showing our semi-natural sowing reciprocal experiment. The bigger picture presents a general overview, centered in one experimental plot. This picture is complemented with four insets in its corners. Each inset shows different growing conditions for seedlings. On the left side, Coca *site*; and on the right, Calderona *site*.
