# Supplementary material for: The Role of Population Origin and Microenvironment in Seedling Emergence and Early Survival in Mediterranean Maritime Pine (Pinus pinaster Aiton)
Source: PLoS One. 2014 Oct 6;9(10):e109132. doi: 10.1371/journal.pone.0109132 (PMC4186868; doi:10.1371/journal.pone.0109132)
Supplement: Table S1 — Log-rank tests to assess heterogeneous patterns in Kaplan-Meier (KM) functions for non-emergence and early survival probability: (i) site-microenvironment combinations for each origin, Coca or Calderona (see Figure 2); and (ii) origin-microenvironment combinations in Calderona site (only for survival, see Figure 3). (PDF) [file pone.0109132.s005.pdf]

Supporting Table S1

Supporting Tables and Figures

The role of population origin and microenvironment in seedling emergence and early survival in Mediterranean maritime pine (*Pinus pinaster* Aiton)

Natalia Vizcaíno-Palomar, Bárbara Revuelta-Eugercios, Miguel A. Zavala, Ricardo Alía, Santiago C. González-Martínez*

*To whom correspondence should be addressed. E-mail: santiago@inia.es

Table S1. Log-rank tests to assess heterogeneous patterns in Kaplan-Meier (KM) functions for non-emergence and early survival probability: (i) site-microenvironment combinations for each origin, Coca or Calderona (see Figure 2); and (ii) origin-microenvironment combinations in Calderona site (only for survival, see Figure 3).

Recruitment stage	KM functions	χ^2	df	*P* values
(i)				
Emergence	Coca *origin*	343	3	< 0.001
	Calderona *origin*	473	3	< 0.001
Survival	Coca *origin*	172	3	< 0.001
	Calderona *origin*	52.9	3	< 0.001
(ii)				
Survival	Calderona *site*	208	3	< 0.001
