# Supplementary material for: The Role of Population Origin and Microenvironment in Seedling Emergence and Early Survival in Mediterranean Maritime Pine (Pinus pinaster Aiton)
Source: PLoS One. 2014 Oct 6;9(10):e109132. doi: 10.1371/journal.pone.0109132 (PMC4186868; doi:10.1371/journal.pone.0109132)
Supplement: Table S2 — Female reproductive success measures for each mother tree. a) Mother trees from Calderona origin. b) Mother trees from Coca origin. NA: Missing data. (PDF) [file pone.0109132.s006.pdf]

Supporting Table S2

Supporting Tables and Figures

The role of population origin and microenvironment in seedling emergence and early survival in Mediterranean maritime pine (*Pinus pinaster* Aiton)

Natalia Vizcaíno-Palomar, Bárbara Revuelta-Eugercios, Miguel A. Zavala, Ricardo Alía, Santiago C. González-Martínez*

*To whom correspondence should be addressed. E-mail: santiago@inia.es

Table S2. Female reproductive success measures for each mother tree. a) Mother trees from Calderona *origin*. b) Mother trees from Coca *origin*. NA: Missing data.

a) Calderona *origin*

N	Candidate mother ID	Relative fertility success
1	M-CA1631	1.165
2	M-CA1632	0.636
3	M-CA1633	1.106
4	M-CA1649	1.575
5	M-CA1650	0.895
6	M-CA1766	0.618
7	M-CA1767	1.220
8	M-CA1768	0.951
9	M-CA1769	0.545
10	M-CA1770	0.231
11	M-CA1771	1.208
12	M-CA1782	1.107
13	M-CA1783	2.035
14	M-CA1785	0.809
15	M-CA1787	1.435
16	M-CA957	0.852
17	M-CA959	1.182
18	M-CA960	1.635
19	M-CA961	0.835
20	M-CA964	0.518
21	M-CA966	1.115
22	M-CA992	1.368
23	M-CA993	0.271
24	M-CA998	0.686
25	M-CA000	NA

b) Coca *origin*

N	Candidate mother ID	Relative fertility success
1	M-CO10	0.633
2	M-CO105	1.042
3	M-CO11	0.813
4	M-CO111	3.400
5	M-CO165	0.243
6	M-CO169	0.269
7	M-CO21	4.195
8	M-CO224	0.054
9	M-CO230	2.439
10	M-CO262	2.204
11	M-CO301	2.233
12	M-CO353	0.906
13	M-CO45	1.501
14	M-CO57	4.171
15	M-CO73	1.490
16	M-CO84	0.469
17	M-COE	1.380
18	M-COI	0.557
19	M-COH	NA
20	M-CO88	NA
21	M-COX1	NA
22	M-COX4	NA
23	M-COX3	NA
24	M-CO110	NA
25	M-COX2	NA
