# Supplementary material for: The Role of Population Origin and Microenvironment in Seedling Emergence and Early Survival in Mediterranean Maritime Pine (Pinus pinaster Aiton)
Source: PLoS One. 2014 Oct 6;9(10):e109132. doi: 10.1371/journal.pone.0109132 (PMC4186868; doi:10.1371/journal.pone.0109132)
Supplement: Text S1 — Effect sizes (Hedges' g*) for local and microenvironmental adaptation. (PDF) [file pone.0109132.s007.pdf]

Supporting Text S1

Effect sizes (Hedges' g) for local and microenvironmental adaptation*

The role of population origin and microenvironment in seedling emergence and early survival in Mediterranean maritime pine (*Pinus pinaster* Aiton)

Natalia Vizcaíno-Palomar, Bárbara Revuelta-Eugercios, Miguel A. Zavala, Ricardo Alía, Santiago C. González-Martínez*

*To whom correspondence should be addressed. E-mail: santiago@inia.es

Effect sizes (Hedges' g) for local and microenvironmental adaptation*

We computed a Hedges' *g** index, a standardized and unbiased estimate of mean differences (see Eq. 1 and 3 below), to assess the magnitude of local adaptation across sites and microenvironments for both emergence and early survival. In our implementation, positive values indicate a better performance of local origin when compared to non-local. Briefly, the difference of average frequencies (*d*) of emergences and/or early survival cases for seedlings with local or foreign origin was calculated along the course of the experiment, *i*, for each combination of site and microenvironment as follows:

$$d_i = \frac{\bar{x}_1 - \bar{x}_2}{s^*} \quad \text{[Eq. 1]},$$

where d_i is the effect size at the *i* time; $\bar{x}_1$ and $\bar{x}_2$ are the average frequency of emergence or early survival cases by experimental plot for, respectively, local and foreign seedlings; and s^* is the adjusted standard deviation:

$$s^* = \sqrt{\frac{(n_1 - 1)s_1^2 + (n_2 - 1)s_2^2}{n_1 + n_2 - 2}} \quad \text{[Eq. 2]},$$

where n_1 and n_2 are the samples sizes for local and foreign origins, and s_i the standard deviation of each origin within a specific environment and site. The effect size estimate d_i has a small upward bias that is relatively large when sample sizes are less than 20. This can be removed using a correction formula (which includes the correction factor, *J*) to derive the unbiased estimate of Hedges' *g**:

$$g^* = J(n_1 + n_2 - 2) \times d_i \approx 1 - \left(\frac{3}{4(n_1 + n_2) - 9} \right) \times d_i \quad \text{[Eq. 3]},$$

and the variance of *g**:

$$v_{g^*} = J^2 v_{di} \quad \text{[Eq. 4]},$$

with the variance of d_i being:

$$v_{di} = \frac{n_1 + n_2}{n_1 \times n_2} + \frac{d_i^2}{2(n_1 + n_2)} \quad \text{[Eq. 5]},$$

Hedges' *g** index was computed using the *compute.es* package in the R environment.

Overall, Calderona *origin* clearly outperformed Coca *origin* for emergence in both sites, and also under different light regimes along the whole course of the experiment. However, Hedges' *g** effect sizes were small (maximum of 0.311), with Hedges' *g** (variance) at the end of the study period of -0.256 (2.016×10^{-4}) and -0.239 (2.014×10^{-4}) for open and closed canopy in Coca, respectively, and of 0.080 (2.002×10^{-4}) and 0.311 (2.024×10^{-4}) in Calderona (see Table below). For survival in Calderona (the only site with survivors), Calderona *origin* outperformed Coca *origin* under closed canopy but not in open environments, as also evidenced in the survival models (see main text). In this case, Hedges' *g** (variance) at the end of the study period was substantially larger than for emergence: -0.442 (6.473×10^{-3}) for open canopy and 0.363 (3.853×10^{-3}) for closed canopy.

Table. Effect sizes (unbiased Hedges' *g** and its variance) for local vs. non-local performance by site (Coca and Calderona) and microenvironment (open- and closed-canopy cover) along the course of the experiment.

	Open canopy		Closed canopy	
	Hedges' *g**	σ^2	Hedges' *g**	σ^2
Days from sowing				
Emergence in Coca				
18	0.321	2.026×10^{-4}	0	0
40	-0.007	2.00×10^{-4}	-0.394	2.039×10^{-4}
60	-0.081	2.001×10^{-4}	-0.276	2.019×10^{-4}
92	-0.265	2.017×10^{-4}	-0.239	2.014×10^{-4}
112	-0.256	2.016×10^{-4}	-0.239	2.014×10^{-4}
Emergence in Calderona				
27	-0.485	2.059×10^{-4}	0.379	2.036×10^{-4}
49	0.107	2.003×10^{-4}	0.787	2.155×10^{-4}
73	0.084	2.002×10^{-4}	0.208	2.011×10^{-4}
99	0.0800	2.001×10^{-4}	0.311	2.024×10^{-4}
Days of survival				
Early survival in Coca				
20	-0.257	1.785×10^{-2}	-0.245	1.671×10^{-2}
52	0	1.771×10^{-2}	-0.142	1.663×10^{-2}
90	0.165	1.664×10^{-2}	-0.071	1.660×10^{-2}
140	0	0	0	0
Early survival in Calderona				
27	0.047	6.321×10^{-3}	0.433	3.879×10^{-3}
45	0.057	6.322×10^{-3}	0.682	4.009×10^{-3}
85	-0.091	6.326×10^{-3}	0.618	3.969×10^{-3}
120	-0.436	3.862×10^{-3}	0.288	3.830×10^{-3}
140	-0.442	6.473×10^{-3}	0.363	3.853×10^{-3}

A positive effect size indicates better performance of local population compared to foreign population in a specific microenvironment at a given site.
